# Equity in patient experiences of primary care in community health centers using primary care assessment tool: a comparison of rural-to-urban migrants and urban locals in Guangdong, China

**DOI:** 10.1186/s12939-018-0758-4

**Published:** 2018-04-27

**Authors:** Chenwen Zhong, Li Kuang, Lina Li, Yuan Liang, Jie Mei, Li Li

**Affiliations:** 1 Department of Health Administration, School of Public Health, Sun Yat-sen University, Guangzhou, 510080 China; 2 Department of Family Medicine and Community Health, Case Western Reserve University, Cleveland, OH 44106 USA

**Keywords:** Equity, Patient experiences, Primary care, Rural-to-urban migrants, China

## Abstract

**Background:**

The equity of rural-to-urban migrants’ health care utilization is already on China’s agenda. The Chinese government has been embarking on efforts to improve the financial and geographical accessibility of health care for migrants by strengthening primary care services and providing universal coverage. Patient experiences are equally vital to migrants’ health care utilization. To our knowledge, no studies have focused on equity in the patient experiences between migrants and locals. Based on a patient survey from Guangdong, China, which has a large number of rural-to-urban migrants, our study assessed the equity in the primary care patient experiences between rural-to-urban migrants and urban locals in the same health insurance context, since different forms of insurance can affect the patient experiences of primary care.

**Methods:**

We stratified our samples by different insurance types into three layers. We assessed primary care patient experiences using a validated Chinese version of the Primary Care Assessment Tool (PCAT), including eight primary care attributes. A ‘PCAT total score’ was calculated. Data were collected through face-to-face and one-on-one surveys in 2014. Propensity score matching (PSM) was used for each layer to generate comparable samples between rural-to-urban migrants and urban locals. Based on the matched dataset, a t-test was employed to compare the primary care patient experiences of the two groups.

**Results:**

Using PSM, 220 patients in the rural-to-urban migrants group were matched to 220 patients in the urban locals group. After the matching, the observed confounding variables were balanced, and the PCAT scores were almost equal between the two groups. The only slight differences existed in the Urban Employee Basic Medical Insurance layer and in the without basic medical insurance coverage layer.

**Conclusions:**

Equity in the primary care patient experiences between rural-to-urban migrants and urban locals seems to have been achieved to some extent. However, there is room for improvement in the equity of coordination of care and comprehensiveness. Policy makers should consider strengthening these two dimensions by integrating the health care system. More attention should be focused on helping migrants break down language and cultural barriers and improving the patient-physician communication process.

**Electronic supplementary material:**

The online version of this article (10.1186/s12939-018-0758-4) contains supplementary material, which is available to authorized users.

## Background

Health equity refers to an absence of disparities in health care or its socio-determinants between groups within different socioeconomic classes or resulting from social, political, economic or other factors that might have an effect on health care status and equity [1–3]. An important social determinant of health care in China is the long existing household registration (‘hukou’) system, which was implemented in 1950s and categorizes people into urban and rural [4] groups; this is a very strong determinant of the rights and privileges affecting socioeconomic wellbeing [5]. When rural people migrate to search for jobs in urban areas, it is never easy for them to convert their household registration to an official urban residency [6]. Since China’s public policies have long been introduced based on this ‘hukou’ system, rural-to-urban migrants have always been identified as being vulnerable due to their poor living conditions [7], lack of social support [8], lack of health risk awareness [9], high medical costs [10, 11] and limited access to health care [12], jobs and insurances [13, 14]. In 2015, the number of migrants in China had reached approximately 247 million, which accounts for 18% of the total Chinese population [15]. Guangdong Province in southeastern China accounts for a large amount of migration, especially in the several cities in the Pearl River Delta region [16]. The increasing number of rural-to-urban migrants and their perception of the fairness of the health care system has caused a great deal of concern for most governments and citizens.

To decrease the disparities between rural-to-urban migrants and urban locals, China has been embarking on health care reform to provide equal access and affordable healthcare for all by 2020 [17]. One of the important measures is the strengthening of primary care, starting with increasing the number of and funding for community health centers (CHCs) in urban areas to make health care services more accessible and less expensive [18]. Being the most financially and geographically accessible approach to health care, primary care provides access to rural-to-urban migrants to obtain health care and reduces the socioeconomic and geographic disparities among different groups [19, 20]. Another goal of China’s current healthcare reform is to establish universal insurance coverage for the whole population. Since the New Rural Cooperative Medical System has been merged into the Urban Resident Basic Medical Insurance in most developed areas, including in Guangdong Province since 2014 [21], the current health care system includes Urban Resident Basic Medical Insurance (URBMI) and Urban Employee Basic Medical Insurance (UEBMI). Financed by employers and employees, migrant workers employed in urban formal sectors, including state-owned, collective, private enterprises and NGOs, are eligible for UEBMI. In some cities, UEBMI also covers part-time workers. The funds are managed to cover outpatient and inpatient services [22]. Migrants who work in urban informal sectors or who are unemployed or self-employed can enroll in URBMI on an individual basis, which is financed mainly by individuals, with few government subsidies. URBMI covers hospital care and catastrophic illness [23]. Evidence has shown that medical health insurance provides positive financial protection for migrants regardless of the type of scheme [22, 24].

All of these efforts can improve patients’ utilization of the health care system by improving geographical and financial access. Furthermore, the patient experience is another important determinant of health care utilization; it may receive comparatively less attention in the literature but is of central importance to patients’ future health care-seeking behavior. According to The Beryl Institute, patient experience is defined as the sum of all interactions shaped by an organization’s culture that influence patient perceptions across the continuum of care [25]. Evidence has shown that how patients perceive the process of consultation may influence their future decisions about seeking care [26]. Saether and colleagues [27] investigated migrants’ access to antiretroviral therapy in Thailand and found that participants experienced discrimination such as rude doctors and challenges accessing health care a second time. Another study in Poland found that when migrants felt a general sense of insecurity about the host area’s health care system, either due to misunderstandings or feeling disregarded by the doctors for being migrants, they might change their health care-seeking strategy, stop visiting doctors in the host area and try to consult doctors in their familiar homeland [28]. In other words, even though equity in financial and geographical accessibility has been achieved, equity in the patient experience could be a significant factor influencing patients’ health care utilization.

We are aware that patient experiences in primary care can be contextualized to measure primary care’s five core dimensions (first-contact utilization, first-contact accessibility, continuity, coordination, and comprehensiveness) and three derivative dimensions (family centeredness, community orientation, and cultural competence). This approach has been widely used in recent years, because it directly describes the primary care process [29–32]. First-contact utilization measures the extent to which primary care acts as an entry point for other levels of care, whereas first-contact accessibility measures the extent to which patients are able to access primary care for each new problem. Continuity of care refers to the longitudinal use of a usual source of care over time. Coordination of care is the linking of health care visits and services between different levels of care so that patients can receive appropriate care and meet health needs. Comprehensiveness of care refers to the availability of a wide range of services in primary care and their appropriate provision. Family centeredness reflects the participation of family in the assessment and treatment of a patient. Community orientation measures providers’ knowledge of community health care needs. Cultural competence measures whether the patient recommends the primary care provider to others [33, 34]. In China, rural-to-urban migrants experience less satisfaction in terms of the types of drugs available, attitudes of health workers, and waiting times than local residents in community health centers [35]. Their experiences of primary care did not show higher scores in any of the domains mentioned above in CHCs than in tertiary hospitals [36].

Studies have focused on equity in primary care experiences between groups with different levels of social disadvantage, such as health status [37], household income [38], age [39], race and ethnicity [40], and disability [41]. People with high self-ratings of health scored higher in the family/community orientation dimension and total primary care scores [37]. The higher household income group was more likely to experience better primary care, such as comprehensiveness [38]. Older individuals reported significantly better experiences across many dimensions of primary care, such as first-contact utilization and continuity [39]. However, no studies have focused on equity in patient experiences of primary care between rural-to-urban migrants and urban locals. Using a patient perception primary care assessment survey [the Primary Care Assessment Tool (PCAT)] administered in 2014, we aimed to determine whether the patient experience of primary care is equal between rural-to-urban migrants and urban locals.

Since medical health insurance can influence patients’ health seeking behavior and affect patients’ health care utilization [42, 43], we first stratified our data into three layers based on the different medical health insurance conditions, including UEBMI, URBMI and individuals without basic medical insurance coverage (WBMI). According to our previous studies, patients’ socioeconomic characteristics have a significant impact on their primary care experiences [44], which may limit the extent to which household registration results in equal access to health care. To adjust for confounding variables and improve causal inferences, we conducted propensity score matching (PSM) within each insurance layer to generate comparable samples between the rural-to-urban migrants and urban locals. PSM has been widely used in primary care observational studies [45–47]. After matching, we compared the patient experiences of the rural-to-urban migrants to the urban locals within each layer. It is also hoped that policy implications regarding the management of issues related to rural-to-urban migrants can be drawn from the analysis.

## Methods

### Study design

This study was a cross-sectional survey conducted in Guangdong Province, southern China, due to its relatively large and diverse population. A stratified, three-stage sampling approach was used to determine the study sample. In the first stage, we selected three cities (Guangzhou, Dongguan, and Shenzhen), which were the most popular metropolitan areas and attracted millions of rural laborers to work as nonpermanent migrants in the Pearl River Delta region in southern China [48]. In the second stage, eight CHCs were selected typically on the basis of their representativeness in terms of the socio-demographic, environmental, and health coverage factors in the area. In the third stage, patients in each CHC were selected by convenience sampling to participate in our survey. Patients who were 18 years or older, could speak Mandarin or Cantonese, and visited the same general practitioners at least three times were selected, since they were considered to have a better understanding of primary care services. Those who were visiting general practitioners were excluded. Based on the standard sample size formula for a cross-sectional study, a target sample size of 680 was set for each group given a type I error of 0.05, type II error of 0.1, and refusal rate of 10% [49].

### Instrument

We used a validated Chinese version of the PCAT to evaluate patient primary care experiences and service delivery [49–51]. Following the original edition of the PCAT [33], an assessment of each subdomain was obtained from the survey subjects’ responses to each question using a 4-point Likert-type scale of 1 (definitely not), 2 (probably not), 3 (probably), and 4 (definitely). Additional response options included ‘not sure’ or ‘don’t know’, which were coded as the middle score (2.5). A ‘PCAT total score’ was created by summing the scores of the eight essential dimensions to summarize the overall primary care experience. Our questionnaire consisted of 42 items. A total of 23 items were used to assess the five core dimensions and three derivative dimensions of primary care. Two items were used to identify whether the patients has usual sources of care, two items measured the frequency of visits to primary care practitioners, one item assessed the patients’ global satisfaction with their current health care provider, and the remaining items were mainly used to reflect the patients’ socioeconomic characteristics, including gender, age, occupation, education, income, marital status, health status, chronic disease status, and social medical insurance status.

### Data collection

Data collection began in June and continued to August 2014. Postgraduate students from Sun Yat-Sen University were hired as interviewers and had at least 6 months’ prior experience working as interviewers. All interviewers were trained by the principal investigator (LK), who was involved in the pilot study, to assist patients to complete the questionnaires, and the interviewers had daily supervision from senior researchers (YL and JM). Individual face-to-face interviews were conducted in the waiting area. The selected patients were asked for permission to participate in our study with a full explanation of the purpose and were told that the survey would not influence their GP visits. After the interview, a small gift was given to patients who took part in the survey to show our gratitude for completing the questionnaire.

### Data analysis

The final sample recruited consisted of 1461 patients. First, we stratified our data into three layers based on the different medical health insurance conditions, including UEBMI (locals: 506, migrants: 121), URBMI (locals: 190, migrants: 334), and WBMI (locals: 19, migrants: 291). Then, we employed PSM within each insurance layer through a nearest neighbor matching algorithm with a match tolerance of 0.1. Finally, we obtained 220 pairs of samples after matching (120 pairs for UEBMI, 82 pairs for URBMI, and 18 pairs for WBMI).

Continuous variables were reported as the mean ± standard deviation, and categorical variables were reported as frequency (%). A chi-square test was used to study any differences between the two groups of categorical data. Based on the matched dataset, the PCAT scores were compared with respect to household registration within each layer using a t-test. The level of significance was *p* < 0.05. All statistical analysis was performed using the statistical package IBM SPSS23.

## Results

### Descriptive statistics

A total of 1461 participants were eligible for inclusion in our study. A total of 220 patients in the rural-to-urban migrant group were matched with 220 patients in the urban local group after propensity score matching. Table 1 shows the characteristics of the entire study sample for urban locals and rural-to-urban migrants before and after the PSM. Before the PSM, 51% of the respondents were rural-to-urban migrants. In both groups, most respondents were female, retired and unemployed. Though the majority of respondents in both groups were 31 to 60 years old, the percentage of rural-to-urban migrants aged > 60 was only 5.0% while for urban locals it was 43.3%. A majority of the respondents stated that their health status was fair or poor (71.8% of the rural-to-urban migrants and 82% of the urban locals). While 60.6% of the urban locals had at least one chronic disease, 74% of the rural-to-urban migrants were without chronic diseases. Most respondents in both groups had not contracted with a primary care physician (PCP). Of the rural-to-urban migrant respondents, 39% were without medical insurance. Compared to the urban locals (only 2.6% of whom were without medical insurance), this proportion is very high.

**Table 1:** Comparability of socioeconomic characteristics and health care utilization patterns by group before and after PSM

	Before PSM (*N* = 1461)			After PSM (*N* = 440)		
	Locals *N*(%)	Migrants *N*(%)	X^2^ significance	Locals *N*(%)	Migrants *N*(%)	X^2^ significance
Sample size	715 (48.9)	746 (51.1)		220 (50)	220 (50)	
Gender			0.158			0.436
Male	263 (36.8)	311 (41.7)		102 (46.4)	94 (42.7)	
Female	451 (63.1)	434 (58.2)		117 (53.2)	126 (57.3)	
Age (years)			*P* < 0.001**			0.063
< 31	57 (8.0)	260 (34.9)		40 (18.2)	57 (25.9)	
31–60	348 (48.7)	449 (60.2)		154 (70.0)	147 (66.8)	
> 60	310 (43.3)	37 (5.0)		26 (11.8)	16 (7.3)	
Occupation			*P* < 0.001**			0.501
Employed	223 (31.2)	173 (23.2)		165 (75)	171 (77.7)	
Retired or unemployed	492 (68.8)	573 (76.8)		55 (25)	49 (22.3)	
Education			*P* < 0.001**			0.556
Primary school or below	122 (17.1)	93 (12.5)		27 (12.3)	20 (9.1)	
Middle/high school	392 (54.8)	515 (69.3)		118 (53.6)	123 (55.9)	
Bachelor’s degree or above	197 (27.6)	135 (18.2)		75 (34.1)	77 (35.0)	
Income			0.333			0.917
< 5000	215 (30.1)	244 (32.7)		63 (28.6)	60 (27.3)	
5000–10,000	255 (35.7)	240 (32.2)		74 (33.6)	73 (33.2)	
> 10,000	245 (34.3)	262 (35.1)		83 (37.7)	87 (39.5)	
Marital status			*P* < 0.001**			0.643
Unmarried	34 (4.8)	88 (11.8)		22 (10)	25 (11.4)	
Married	681 (95.2)	658 (88.2)		198 (90)	195 (88.6)	
Health status			*P* < 0.001**			0.679
Fair or poor	586 (82)	536 (71.8)		155 (70.5)	151 (68.6)	
Very good or good	129 (18)	210 (28.2)		65 (29.5)	69 (31.4)	
Chronic diseases			*P* < 0.001**			0.302
No	282 (39.4)	552 (74)		147 (66.8)	157 (71.4)	
Yes	433 (60.6)	194 (26)		73 (33.2)	63 (28.6)	
Number of CHC visits in the last year			*P* < 0.001**			0.093
< 6	270 (37.8)	522 (70)		121 (55)	143 (65.0)	
6–30	377 (52.7)	214 (28.7)		91 (41.4)	72 (32.7)	
> 30	68 (9.5)	10 (1.3)		8 (3.6)	5 (2.3)	
Contracted with PCP			*P* < 0.001**			1.000
Yes	96 (13.4)	35 (4.7)		19 (8.6)	17 (7.7)	
No	619 (86.6)	711 (95.3)		201 (91.4)	203 (92.3)	
Social medical insurance			*P* < 0.001**			1.000
Basic medical insurance systems for urban workers	506 (70.8)	121 (16.2)		120 (54.5)	120 (54.5)	
Basic medical insurance systems for residents	190 (26.6)	334 (44.8)		82 (37.3)	82 (37.3)	
Without medical insurance	19 (2.6)	291 (39)		18 (8.2)	18 (8.2)	

Note:1. *N* number of patients; *p < 0.05; **p < 0.01; *PSM* propensity score matching, *PCP* primary care physician

2. Differences were explored using the chi-square test between urban locals (with ‘hukou’) and rural-to-urban migrants (without ‘hukou’) who settled permanently or temporarily somewhere other than the original household registration location before and after propensity score matching

There were statistically significant differences in age, employment status, education, marital status, health status, chronic disease condition, number of CHC visits in the last year, whether they had contracted with a PCP or not and social medical insurance between the two groups before the PSM (*p* < 0.05, Table 1). We found that after matching all the confounding variables observed, the patient characteristics in each layer were balanced (*p* > 0.05). An additional file shows this in greater detail [see Additional file 1].

### Propensity score matching (PSM) results

**Fig. 1:**
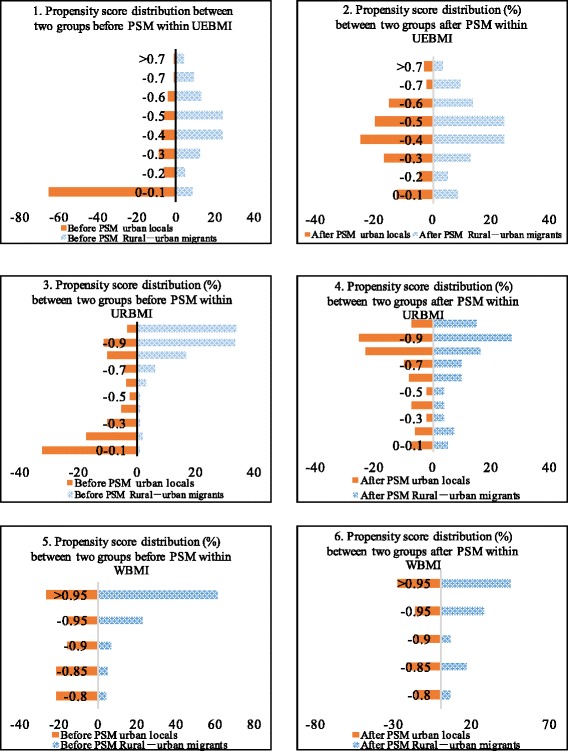
Distributions of the propensity scores between two groups before and after matching within each layer

The propensity score was constructed using common logistic regression modeling in which potential confounding variables were considered independent variables, including gender, age, employment status, household income, education, marital status, health status, chronic disease status of respondents, the number of community health center visits in the last year and whether respondents had contracted with a PCP or not. The group assignment (rural-to-urban migrants vs. urban locals) was included as the dependent variable. Figure 1 shows the distribution of propensity scores between the two groups before and after matching within the three layers. We can easily determine that after the PSM, the distribution of biases and confounding variables between the two groups was almost balanced.

### Primary care dimension scores

Table 2 presents the primary care assessment scores between migrants and urban locals in each insurance layer respectively, before and after the PSM. The total scores of rural-to-urban migrants in different insurance statuses ranged from 13.63 to 13.68, while the scores of urban locals ranged from 13.47 to 13.49 before the PSM. After the matching, while other scores may have had small fluctuations, only the score of migrant patients who had no health insurance coverage changed significantly from 13.64 to 12.63.

**Table 2:** Analysis of the PCAT scores between the two groups within each layer before and after PSM

	Before PSM									After PSM								
	UEBMI Mean (Sd.)			URBMI Mean (Sd.)			WMI Mean (Sd.)			UEBMI Mean (Sd.)			URBMI Mean (Sd.)			WMI Mean (Sd.)		
	Locals	Migrants	*P*	Locals	Migrants	*P*	Locals	Migrants	*P*	Locals	Migrants	*P*	Locals	Migrants	*P*	Locals	Migrants	*P*
First-contact utilization	2.61 (0.60)	2.64 (0.76)	0.608	2.89 (0.68)	3.31 (0.70)	< 0.001**	2.88 (0.58)	2.72 (0.70)	0.334	2.67 (0.67)	2.64 (0.76)	0.731	3.03 (0.74)	3.14 (0.77)	0.363	2.87 (0.60)	2.60 (0.88)	0.293
First-contact accessibility	1.66 (0.37)	1.76 (0.39)	0.006**	1.58 (0.37)	1.61 (0.39)	0.427	1.63 (0.35)	1.72 (0.37)	0.340	1.71 (0.38)	1.76 (0.39)	0.281	1.61 (0.38)	1.62 (0.40)	0.894	1.63 (0.36)	1.56 (0.32)	0.520
Continuity	2.69 (0.81)	2.30 (0.85)	< 0.001**	2.77 (0.85)	2.32 (0.84)	< 0.001**	2.18 (0.93)	2.45 (0.81)	0.158	2.44 (0.79)	2.29 (0.85)	0.171	2.64 (0.86)	2.40 (0.82)	0.077	2.13 (0.94)	2.28 (0.76)	0.607
Comprehen-siveness	1.90 (0.55)	1.82 (0.58)	0.131	1.71 (0.51)	1.71 (0.48)	0.922	1.86 (0.44)	1.72 (0.47)	0.215	1.84 (0.54)	1.82 (0.58)	0.786	1.70 (0.50)	1.69 (0.40)	0.949	1.83 (0.44)	1.56 (0.37)	0.047*
Coordination of care	1.75 (0.46)	1.96 (0.47)	< 0.001**	1.75 (0.41)	2.04 (0.45)	< 0.001**	1.71 (0.37)	1.97 (0.47)	0.018*	1.80 (0.33)	1.96 (0.47)	0.002**	1.89 (0.43)	1.98 (0.41)	0.148	1.70 (0.38)	1.83 (0.38)	0.312
Family centeredness	1.72 (0.72)	1.86 (0.79)	0.053	1.69 (0.70)	1.73 (0.66)	0.587	1.93 (0.90)	1.74 (0.70)	0.244	1.76 (0.69)	1.87 (0.79)	0.234	1.78 (0.72)	1.80 (0.64)	0.863	1.93 (0.93)	1.42 (0.68)	0.083
Community orientation	1.41 (0.45)	1.52 (0.58)	0.029*	1.37 (0.38)	1.42 (0.40)	0.145	1.49 (0.49)	1.45 (0.41)	0.672	1.47 (0.45)	1.52 (0.58)	0.445	1.42 (0.40)	1.43 (0.38)	0.764	1.52 (0.49)	1.42 (0.48)	0.526
Cultural competence	1.86 (0.95)	1.98 (1.02)	0.218	1.97 (0.97)	2.00 (1.02)	0.692	2.05 (1.17)	2.10 (1.00)	0.839	1.93 (0.97)	1.97 (1.02)	0.746	2.12 (1.03)	2.07 (0.99)	0.758	2.11 (1.18)	2.03 (1.09)	0.827
Total score	13.47 (2.73)	13.63 (3.09)	0.555	13.49 (2.57)	13.68 (2.48)	0.425	13.47 (2.89)	13.64 (2.54)	0.781	13.42 (2.73)	13.63 (3.11)	0.573	13.86 (2.82)	13.77 (2.48)	0.816	13.48 (2.97)	12.63 (2.86)	0.392

Sd. Standard deviation, *p<0.05, **p<0.01

Differences were explored by t-test within each layer between urban locals and rural-to-urban migrants

**Fig. 2:**
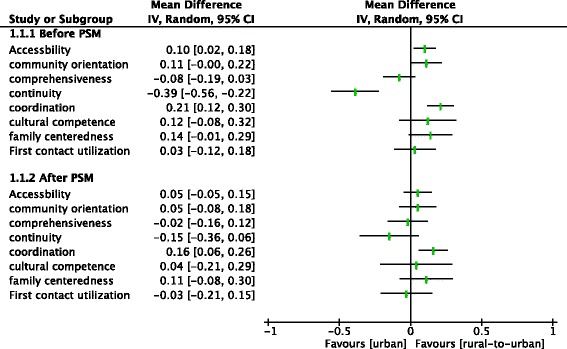
Analysis of the PCAT scores between two groups within the UEBMI before and after PSM

**Fig. 3:**
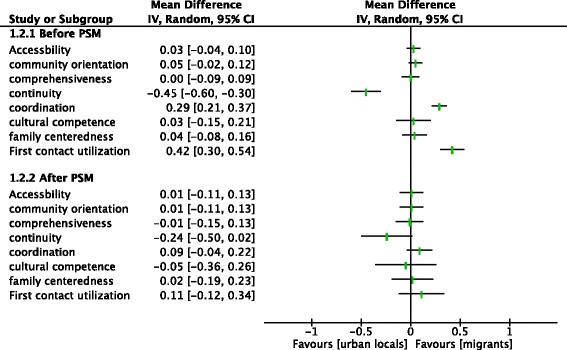
Analysis of the PCAT scores between two groups within the URBMI before and after PSM

**Fig. 4:**
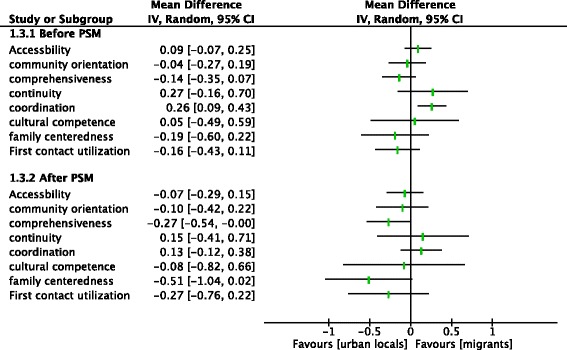
Analysis of the PCAT scores between two groups within the WBMI before and after PSM

After the PSM, the data from the two groups were balanced by socioeconomic characteristics and health service utilization patterns. In the UEBMI layer, rural-to-urban migrants reported slightly higher scores for the attributes of first-contact accessibility, coordination, family centeredness, community orientation, cultural competence and total score. A significant difference was only reported for coordination of care (*p* = 0.002). In the WBMI layer, urban locals reported a higher score for the attribute of comprehensiveness (*p* = 0.047). In the URBMI layer, there turned out to be no difference between rural-to-urban migrants and urban locals (Table 2, Figs. 2, 3 and 4).

## Discussion

### Main finding

In this study, we compared rural-to-urban migrants’ primary care experience to that of urban locals’ in the same insurance context after PSM using a validated Chinese edition of the PCAT. The findings demonstrated that patient primary care experiences between rural-to-urban migrants and urban locals were almost equal within the same health insurance layer after using PSM. However, there was still a small difference in the perception of the coordination of care within the UEBMI layer and the perception of comprehensiveness within the WBMI layer between the two groups.

In our study, the PCAT scores for the eight attributes and the total scores were almost equal for both groups (rural-to-urban migrants and urban locals). This implies that regardless of the household registration with which patients are associated, their experiences of primary care are the same. Li [35] conducted a survey to compare patients’ perceived quality of care among migrants and local patients using patient satisfaction as an indicator and reached a similar conclusion, with no difference in the overall satisfaction between the local residents and migrants. Over the past decades, health care reform has ensured affordable access to health care services with the achievement of nearly universal health insurance coverage and rapidly developing primary care. The results from our study demonstrate that China’s health reform may have to some extent achieved the goal of equity in patients’ experiences of health care between migrants and locals.

Nevertheless, there were still small differences in the UEBMI layer and the WBMI layer between the two groups. Since we have employed PSM to improve the comparability of the two groups, theoretically, these differences were not caused by socioeconomic status but by household registration.

One surprising finding was that the vulnerable rural-to-urban migrants (1.96±0.47) attributed a substantially higher score than the urban locals (1.80±0.33) to the coordination of care within the UEBMI layer. When we then compared the items under coordination of care, we found that among the patients with UEBMI coverage, the item “whether primary physicians would recommend specialists for you when you need a referral” in the coordination domain scored significantly higher than for urban locals (1.74±0.98 for rural-to-urban migrants, 1.29±0.60 for urban locals, *P* = 0.003). In moving from the rural to the urban context, migrants’ social support network was fragmented. Research has shown that migrants consider PCPs to be more familiar with their health condition [36]. When they must seek health care from specialists, they might be prone to go to primary health centers for help. Yang et al. [17] revealed that patients in Chinese urban areas did not generally trust community health service centers. This belief may result in urban patients choosing to see specialists in hospitals when needed, which weakens the use of appropriate referrals by PCPs.

In our study, of the 746 migrants surveyed, 291 had no health insurance. In the local group, only 19 (of 715) patients were not covered by health insurance. These data mainly reflected the fact that migrants were still largely excluded from medical benefits in urban cities. For these patients’ WBMI coverage, urban locals gave higher scores than rural-to-urban migrants to the attribute of comprehensiveness. The comprehensiveness of primary care measures the ability of physicians to provide a wide range of care in response to patients’ health needs [19]. While providing comprehensiveness, the health care provider’s accumulated knowledge of the patient could be directly affected by patient-physician communication [52]. Compared to urban locals, some of the migrants might have trouble communicating to physicians due to language barriers and a lack of family support. Cleland found that physicians might need to make a greater effort to understand migrant patients because of language and cultural barriers [53]. Therefore, for urban locals, language familiarity and a better understanding of the health care system may be important facilitators of their ability to communicate more easily with health service providers and receive more comprehensive care.

### Limitations

There are several limitations to this study. First, our findings might overestimate the real situation of health equity between rural-to-urban migrants and urban locals since our survey was conducted among patients who have consulted at CHCs at least once in a year. Patients who felt discriminated against or who once had an unsatisfactory experience in the CHCs would have been excluded in our study. Second, we did not investigate patient primary care experience in health care settings other than the CHCs, though the CHCs are becoming the major primary care providers in China. Participants could also experience primary care in tertiary hospitals, and so any implications of the findings should be tempered with a word of caution. Third, the sample of local patients without medical health insurance coverage included 19 respondents, which was a relatively small sample. After the PSM, there were only 18 pairs of samples in the WBMI layer, which lacked enough power to detect intervention effects. Despite all of these limitations, to our knowledge, our study is the first in China to address the equity of patient primary care experiences between rural-to-urban migrants and urban locals covered by the same insurance, and it provides significant evidence for health care policy in China.

## Conclusions

Our study demonstrates that rural-to-urban migrants receiving primary care from CHCs reported equal primary care experiences when compared to urban locals in the same medical health insurance context. The hope of health care equity between rural-to-urban migrants and urban locals seems to have been achieved in the CHCs to some extent. However, there is room for improvement in the equity of coordination of care and comprehensiveness. Policy makers should consider strengthening coordination of care and comprehensiveness in primary care by integrating health care systems. More attention should be focused on helping migrants break down language and cultural barriers and improving the patient-physician communication process.

## Additional file

Additional file 1 Comparability of socioeconomic characteristics and health care utilization patterns by group based on different health insurance schemes before and after PSM. (DOCX 30 kb)

## Abbreviations

CHCs: community health centers
PCAT: Primary Care Assessment Tool
PSM: propensity score matching
UEBMI: Urban Employee Basic Medical Insurance
URBMI: Urban Resident Basic Medical Insurance
WBMI: without basic medical insurance coverage
