# Supplementary material for: Equity in patient experiences of primary care in community health centers using primary care assessment tool: a comparison of rural-to-urban migrants and urban locals in Guangdong, China
Source: Int J Equity Health. 2018 Apr 27;17:51. doi: 10.1186/s12939-018-0758-4 (PMC5921537; doi:10.1186/s12939-018-0758-4)
Supplement: Supplementary file 1 — Comparability of socioeconomic characteristics and health care utilization patterns by group based on different health insurance schemes before and after PSM. (DOCX 30 kb) [file 12939_2018_758_MOESM1_ESM.docx]

**Additional file 1 Comparability of socioeconomic characteristics and health care utilization patterns by groups based on different health insurance schemes before and after PSM**

|  | Before PSM | | | | | | | | | After PSM | | | | | | | | |
| --- | --- | --- | --- | --- | --- | --- | --- | --- | --- | --- | --- | --- | --- | --- | --- | --- | --- | --- |
|  | UEBMI N(%) | | | URBMI N(％) | | | WMI N(%) | | | UEBMI N(%) | | | URBMI N(％) | | | WMI N(%) | | |
|  | Locals | Migrants | P | Locals | Migrants | P | Locals | Migrants | P | Locals | Migrants | P | Locals | Migrants | P | Locals | Migrants | P |
| Sample size | 506  (80.7) | 121  (19.3) |  | 190  (36.3) | 334  (63.7) |  | 19  (6.1) | 291  (93.9) |  | 120 | 120 |  | 82 | 82 |  | 18 | 18 |  |
| **Gender** |  |  | **0.048*** |  |  | **0.001** |  |  | **0.886** |  |  | **0.606** |  |  | **0.522** |  |  | **0.674** |
| Male | 193  (38.1) | 58  (47.9) |  | 65  (34.2) | 172  (51.5) |  | 5  (26.3) | 81  (27.8) |  | 61  (50.8) | 57  (47.5) |  | 37  (45.1) | 34  (41.5) |  | 4  (22.2) | 3  (16.7) |  |
| Female | 313  (61.9) | 63  (52.1) |  | 124  (65.3) | 161  (48.2) |  | 14  (73.7) | 210  (72.2) |  | 59  (49.2) | 63  (52.5) |  | 44  (53.7) | 48(58.5) |  | 14  (77.8) | 16(83.3) |  |
| **Age** |  |  | **P<0.001**** |  |  | **P<0.001**** |  |  | **0.097** |  |  | **0.077** |  |  | **0.583** |  |  | **0.884** |
| <31 | 31  (6.2) | 34  (28.1) |  | 21  (11.1) | 103  (30.8) |  | 5  (26.3) | 123  (42.3) |  | 20  (16.7) | 33  (27.5) |  | 15  (18.3) | 19  (23.2) |  | 5  (27.8) | 5(27.8) |  |
| 31-60 | 245  (48.4) | 79  (65.3) |  | 93  (48.9) | 225  (67.4) |  | 10  (52.6) | 145  (49.8) |  | 86  (71.7) | 79  (65.8) |  | 58  (70.7) | 57  (69.5) |  | 10  (55.6) | 11(61.1) |  |
| >60 | 230  (45.5) | 8  (6.6) |  | 76  (40.0) | 6  (1.8) |  | 4  (21.1) | 23  (7.9) |  | 14  (11.7) | 8  (6.7) |  | 9  (11) | 6  (7.3) |  | 3  (16.7) | 2(11.1) |  |
| **Occupation** |  |  | **P<0.001**** |  |  | **P<0.001**** |  |  | **0.033*** |  |  | **0.470** |  |  | **0.861** |  |  | **0.729** |
| Employed | 158  (31.2) | 105  (86.8) |  | 59  (31.1) | 303  (90.7) |  | 6  (31.6) | 126  (43.3) |  | 100  (83.3) | 104  (86.7) |  | 23  (28) | 22  (26.8) |  | 6  (33.3) | 7(38.9) |  |
| Retired or unemployed | 348  (68.8) | 16  (13.2) |  | 131  (68.9) | 31  (9.3) |  | 13  (68.4) | 165  (56.7) |  | 20  (16.7) | 16  (13.3) |  | 59  (72) | 60  (73.2) |  | 12  (66.7) | 11(61.1) |  |
| **Education** |  |  | **0.053** |  |  | **P<0.001**** |  |  | **0.649** |  |  | **0.834** |  |  | **0.395** |  |  | **0.887** |
| Primary school or below | 74  (14.6) | 11  (9.1) |  | 45  (23.7) | 28  (8.4) |  | 3  (15.8) | 54  (18.6) |  | 13  (10.8) | 11  (9.2) |  | 11  (13.4) | 6  (7.3) |  | 3  (16.7) | 3(16.7) |  |
| Middle/high school | 272  (53.8) | 59  (48.8) |  | 107  (56.3) | 249  (74.6) |  | 13  (68.4) | 207  (71.1) |  | 60  (50) | 58  (48.3) |  | 46  (56.1) | 52  (63.4) |  | 12  (66.7) | 13(72.2) |  |
| Bachelor ‘s degree or above | 160  (31.6) | 51  (42.1) |  | 34  (17.9) | 57  (17.1) |  | 2  (15.8) | 27  (9.3) |  | 47  (39.2) | 51  (42.5) |  | 25  (30.5) | 24  (29.3) |  | 3  (16.7) | 2(11.1) |  |
| **Income** |  |  | **0.427** |  |  | **0.014*** |  |  | **0.958** |  |  | **0.626** |  |  | **0.905** |  |  | **0.213** |
| <5000 | 157  (31) | 34  (28.1) |  | 52  (27.4) | 115  (34.4) |  | 6  (31.6) | 95  (32.6) |  | 40  (33.3) | 34  (28.3) |  | 17  (20.7) | 15  (18.3) |  | 6  (33.3) | 11  (61.1) |  |
| 5000-10000 | 196  (38.7) | 43  (35.5) |  | 54  (28.4) | 114  (34.1) |  | 5  (26.3) | 83  (28.5) |  | 43  (35.8) | 43  (35.8) |  | 26  (31.7) | 28  (34.1) |  | 5  (27.8) | 2  (11.1) |  |
| >10000 | 153  (30.2) | 44  (36.4) |  | 84  (44.2) | 105  (31.4) |  | 8  (42.1) | 113  (38.8) |  | 37  (30.8) | 43  (35.8) |  | 39  (47.6) | 3  (47.6) |  | 7  (38.9) | 5  (27.8) |  |
| **Marital status** |  |  | **0.001**** |  |  | **0.015*** |  |  | **0.976** |  |  | **0.678** |  |  | **0.798** |  |  | **1.000** |
| Unmarried | 20  (4.0) | 14  (11.6) |  | 12  (6.3) | 44  (13.2) |  | 2  (10.5) | 30  (10.3) |  | 12  (10) | 14  (11.7) |  | 8  (9.8) | 9  (11.0) |  | 2  (11.1) | 2  (11.1) |  |
| Married | 486  (96) | 593  (88.4) |  | 178  (93.7) | 290  (86.8) |  | 17  (89.5) | 261  (89.7) |  | 108  (90) | 106  (88.3) |  | 74  (90.2) | 73  (89.0) |  | 16  (88.9) | 16  (88.9) |  |
| **Health status** |  |  | **P<0.001**** |  |  | **0.686** |  |  | **0.590** |  |  | **0.330** |  |  | **0.481** |  |  | **0.735** |
| Fair or poor | 426  (84.2) | 80  (66.1) |  | 148  (77.9) | 255  (76.3) |  | 12  (63.2) | 201  (69.1) |  | 86  (71.7) | 79  (65.8) |  | 58  (70.7) | 62  (75.6) |  | 11  (61.1) | 10  (55.6) |  |
| Very good or good | 80  (15.8) | 41  (33.9) |  | 42  (22.1) | 79  (23.7) |  | 7  (36.8) | 90  (30.9) |  | 34  (28.3) | 41  (34.2) |  | 24  (29.3) | 20  (24.4) |  | 7  (38.9) | 4  (44.4) |  |
| **Chronic diseases** |  |  | **P<0.001**** |  |  | **P<0.001**** |  |  | **0.394** |  |  | **0.168** |  |  | **0.609** |  |  | **0.248** |
| No | 185  (36. 6) | 87  (71.9) |  | 81  (42.6) | 245  (73.4) |  | 16  (84.2) | 220  (75.6) |  | 76  (63.3) | 86  (71.7) |  | 56  (68.3) | 59  (72) |  | 15  (83.3) | 12  (66.7) |  |
| Yes | 321  (63.4) | 34  (28.1) |  | 109  (57.4) | 89  (26.6) |  | 3  (15.8) | 71  (24.4) |  | 44  (36.7) | 34  (28.3) |  | 26  (31.7) | 23  (28) |  | 3  (16.7) | 6  (33.3) |  |
| **Number of CHC visits in last year** |  |  | **P<0.001**** |  |  | **P<0.001**** |  |  | **0.181** |  |  | **0.190** |  |  | **0.329** |  |  | **0.137** |
| <6 | 177  (35) | 74  (61.2) |  | 82  (43.2) | 232  (69.5) |  | 11  (57.9) | 216  (74.2) |  | 59  (49.2) | 73  (60.8) |  | 51  (62.2) | 55  (67.1) |  | 11  (61.1) | 15  (83.3) |  |
| 6-30 | 278  (54.9) | 42  (34.7) |  | 92  (48.4) | 101  (30.2) |  | 7  (36.8) | 71  (24.4) |  | 55  (45.8) | 42  (35.0) |  | 29  (35.4) | 27  (32.9) |  | 7  (38.9) | 3  (16.7) |  |
| >30 | 51  (10.1) | 5  (4.1) |  | 16  (8.4) | 1  (0.3) |  | 1  (5.3) | 4  (1.4) |  | 6  (5.0) | 5  (4.2) |  | 2  (2.4) | 0 |  | 0 | 0 |  |
| **Contracted with PCP** |  |  | **0.067** |  |  | **0.534** |  |  | **0.527** |  |  | **0.540** |  |  | **0.468** |  |  | **0.310** |
| Yes | 84  (16.6) | 12  (9.9) |  | 11  (5.8) | 15  (4.5) |  | 1  (5.3) | 8  (2.7) |  | 15  (12.5) | 12  (10) |  | 3  (3.7) | 5  (6.1) |  | 1  (5.6) | 0 |  |
| No | 422  (83.4) | 109  (90.1) |  | 179  (94.2) | 319  (95.5) |  | 18  (94.7) | 283  (97.3) |  | 105  (87.5) | 108  (90) |  | 79  (96.3) | 77  (93.9) |  | 17  (94.4) | 18  (100) |  |

Notes: 1. UEBMI= Urban Employee Basic Medical Insurance; URBMI= Urban Residents Basic Medical Insurance; WBMI= without medical insurance covered.

2. Differences were explored by chi-square test between urban locals (with ‘hukou’) and rural-to-urban migrants (without ‘hukou’) who have settled permanently or temporarily somewhere other than the original household registration place before and after propensity score matching within each layer.
